# New York Odontological Society

**Published:** 1893-06

**Authors:** 

**Affiliations:** New York Odontological Society


### Reports of Society Meetings.

### NEW YORK ODONTOLOGICAL SOCIETY.

A REGULAR meeting of the New York Odontological Society was held on Tuesday evening, March 21, 1893, at the New York Academy of Medicine, No. 17 West Forty-third Street, New York City, Dr. Woodward presiding.

### INCIDENTS OF OFFICE PRACTICE.

*Dr. Francis.*—I hold here an appliance which I was requested to exhibit this evening. It is a lens that can be held attached to the finger. It is double-jointed, and can be turned in any desired direction. I had it made from a design of Dr. Williams, of Boston, and find it very useful when preparing and filling cervical cavities of the incisors.

I exhibit also an instrument designed as an amalgam plugger. It is very convenient for introducing amalgam, and I value it greatly. Here is another instrument which I once exhibited at a clinic of another society; it is a forceps for clipping the overhanging gum from inferior third molars. It may be slipped under the gum, and in an instant will clip out a small piece of that tissue nicely.

I have an incident of office practice which I think worth relating. A middle-aged lady came to me to seek relief from pain caused by a diseased second inferior bicuspid. Her face as well as her gum was very much swollen, and had been in that condition for some days. The tooth contained an amalgam filling on its posterior surface. I drilled through the pulp-chamber and cleansed the root as well as I was able, using peroxide of hydrogen, which caused bubbles to exude in great profusion. By making applications to the face and gum, I succeeded in reducing the inflammation and so rendered her quite comfortable, but the trouble soon after returned. I treated it again, and it once more seemed all right. In the mean time, she visited Washington and was absent some weeks, but had no trouble while away. This all occurred just previous to the time that Dr. Thomas, of Philadelphia, read a paper before this

Society on the extraction of teeth, and I then decided that if this patient had trouble with her tooth again I would advise its removal. This proved to be the case, and, at my suggestion, she went to Dr. Hasbrouck and had it extracted. He gave her the tooth to show to me. It was necrosed at the apex. There is an incident connected with this that I wish to state. The lady was under treatment for partial deafness. A few days ago she called on me and remarked, "My physician says that my deafness is fifty per cent. less than it was before I had the tooth out." Her aurist felt convinced that the presence of this diseased tooth had aggravated the disease.

I would like to state another incident, although not one of office practice. I want to tell you of a recent visit to Buffalo, that you may know how well favored our brethren are in that city. It has a new medical university, which is remarkably complete in all its appointments, and excellent provisions have been made for instruction in dentistry. In it is a magnificent infirmary as well as a laboratory, and everything is nicely arranged. The students are on the same footing with medical students; they attend many of the lectures together, and have free access to the clinics of the general hospital. I was much pleased with everything I saw there, and so concluded to report to you, to let you know how things are progressing in the western part of the State.

*Dr. Howe.*—I wish to endorse what Dr. Francis has said about the usefulness of the lens, supported on the finger adjustable support devised by our friend, Dr. J. L. Williams. I have one differing somewhat from Dr. Francis's, but made from a description given me by Dr. Williams. While it has some defects, it serves the purpose well.

I have found, in the use of a lens for magnifying work under the instrument, that a relatively low-power lens answers the purpose very well indeed, and has an advantage in not requiring one to be very particular about the focus.

*Dr. Jarvie.*—I am treating a case at present which may be interesting and may help others to diagnose similar conditions. A lady came to me three or four weeks ago with soreness in the right superior lateral and a small fistulous opening near the margin of the gum. The opening was a very fine one; the appearance of the tooth did not indicate a dead pulp, and the symptoms made me think the pulp was not dead; and yet I could not understand why there should be a fistula. The direction of the sinus indicated that it was connected in some way with the lateral. I examined the tooth and could not find decay. With a fine instrument I worked up under the gum as far as I could. On the labial surface the gum clung quite close to the root, but I found when I worked an exploring instrument on the lingual side of the root the point of my explorer dropped into a cavity. I pressed the gum away with cotton, and when I had the cavity sufficiently exposed, I discovered that it was not caused by decay, but by absorption, the cavity reaching to the pulp. I filled the cavity with gutta-percha, hoping that the pulp would give no further trouble, but it did, and I determined to destroy the pulp. To that end I drilled a cavity through the palatine surface of the tooth until I got to the pulp, and then I applied creosote and arsenic. About ten days ago a spot appeared on the labial surface of the gum, about a quarter of an inch in diameter, looking somewhat like the common ulcerated surface that we see sometimes, and yet not exactly like it. The tooth was very sore, and in the centre of that little spot was a fistula. I discovered the other day that I could work through that and into another cavity on the labial surface of the root, also caused by absorption and also reaching to the pulp. The cavity was perfectly round, as though a small worm had burrowed in, but was much larger on the inside than at the opening. I have not explored it thoroughly, but a burnisher that I can just enter at the opening I can move in every direction upon the inside of the cavity. The cavity must be four or five times larger than at the opening. The tooth is perfectly sound so far as decay is concerned, and the pulp is alive. What has caused the absorption on corresponding points, one on the labial surface and one on the palatine surface of the root? I have never met with such a case before.

Subject passed.

The Secretary then read a communication from Dr. Walker, notifying the Society of the death of Dr. Frederick A. Levy, of Orange, N. J., and Dr. W. W. Allport, of Chicago, Ill.

*Dr. Bogue.*—I move a committee be appointed to express the sentiments of the Society.

The President appointed Drs. Bogue, Francis, and Jarvie as such committee, and instructed them to report at the next meeting.

The President then introduced Dr. Albert Westlake, who read a paper on "Correction of Deformities of the Oral Region," which he illustrated with various models and photographs. He also showed models of a case, which he is now treating, in which the ear is deformed.

(For Dr. Westlake's paper, see page 416.)

DISCUSSION.

*Dr. Westlake.*—After Dr. McBurney first operated I took charge. It has a slit a quarter of an inch long close to the skull. I have cast the ear, and have already made an attachment with aluminum, and a gold bar fastened through here holds it right in position. To remove it, a fine instrument is pressed on a point the size of the head of a needle, and it springs off readily. When the enamel is on, I think it will be a perfect adaptation. It was a beautiful surgical operation, and the result is very satisfactory.

*Dr. Jarvie.*—Was the piece detached entirely?

*Dr. Westlake.*—No; I will pass around the model, so you can see. This is the shape of the other ear, but in a smaller size. I have several other interesting cases in hand, including a nose and malar bone.

I have sketched this case, giving a general idea how it is fastened. This one is the case of a young woman who was caught in an elevator. She was treated at Roosevelt Hospital and referred to me by Dr. Towsey. This plate fits very accurately on the bridge of the nose, and is held by improved cement, but the glasses are used to hide the upper-junction glasses.[1] (Figs. 1, 2, and 3.)

When I agreed to read a paper before this Society, I had a number of cases under way, but have not been able to complete them for presentation to-night. One that I had last fall was published in the local papers. The description given was very interesting from a reporter's stand-point, and I expected the gentleman to be here to-night, but he has not come. I would have been pleased to have had him present on account of the practical demonstration of this method of correcting the deformities of the nose. It is a singular thing, but Dr. McBurney said he had never seen any deformity like the one which is represented here in the model, and yet the mother of this boy says she knows of five cases. Three have already written to me requesting a history of the case. I think the process a very simple one, the principal point being, of course, in the question of fastening. Dr. McBurney tried to bring the hair down in order to have it cover some of the defects which would naturally occur. In the daytime they look very natural, showing translucency of flesh. At night the change is hardly perceptible.

*Dr. Howe.*—Is the auditory canal absent in this boy's case?

---

[1] Mr. W. E. Duncan, of E. B. Meyrowitz, opticians, aided me in the adjustment of the special style of glasses needed.

*Dr. Westlake.*—Yes; there is no auditory canal whatever; there is a slight depression here, which I suspected might lead to an internal canal, but I found that the boy could not hear a thing by closing the other ear. When I placed a watch back of the ear, he could hear it, but that was by contact only.

*Dr. Ottolengui.*—I think that almost any appliance of a mechanical nature, constructed to hold an artificial organ, will eventually become useless, or at least inadequate. We can hinge a door to a house, but mechanical attachments to soft tissue is a totally different matter. Any impingement will produce absorption, resulting in an alteration of the soft tissue, which must modify the fit of the appliance. Dr. Westlake truly says that the problem of attaching the organ is the greatest difficulty. I therefore desire to narrate the history of a case which will prove instructive in this connection.

About three years ago a gentleman came from Europe to have Dr. Kingsley make him an obturator and a nose, the necessity for both having its origin in a syphilitic lesion. Dr. Kingsley hesitated to undertake the nose because of the difficulty of attachment, but the gentleman had already solved that problem for himself. He was at the time wearing a nose which had been made for him in Europe, which, though unsatisfactory from an artistic and æsthetic point of view, was nevertheless held firmly in place. The statement which I am about to make may be astonishing, but it is nevertheless true. This man used a sort of glue, or cement, for attaching his artificial noses. I cannot at this moment give the precise formula, but it is composed of tragacanth and one or two other of the sticky gums. Dr. Kingsley made for this man six noses, which he paints to suit climatic changes and effects of daylight and gaslight.

*Dr. Littig.*—I had the pleasure of seeing the finished case that was mentioned by Dr. Westlake, and I must say that the appearance and adaptation were as complete as I have ever seen. In fact, I do not doubt but that the party might walk into the room and a casual observer would not notice that the nose was artificial. The attachment was complete in the fact that there was no movement during mastication, it being attached to the plate, and the plate being fastened to the teeth, so it would hold perfectly firm. That the tissues may yield in the course of time I have no doubt; but the attachment that Dr. Westlake has with the spring, which will shut down closely, so as to hold the rod which fastens through the plate, I think will enable the patient to wear it a considerable length of time without alteration.

Dr. J. Adams Bishop then read a paper on "Experiences in treating Cleft Palates."

(For Dr. Bishop's paper, see page 406.)

*Dr. Bishop.*—I wish to say a word or two as an addenda to my paper of last month on fractures of the lower jaw. I felt that our colleges and our schools were not educating young men to treat that bone as it should be treated. The next week the *New York Medical Journal* had an article entitled "The Treatment of Fractures," by George W. King, M.D., of Montana. I will read one or two paragraphs from the paper:

"To know all the steps of an operation is one thing; to execute them in a masterly manner is another. Special training for the work is absolutely demanded in either case. There are very few cases strictly surgical that do not require the services of the hands as well as of the head.

"Division of labor is therefore an advantage, in that greater skill may be acquired by those whose work is limited to certain lines of practice. Naturally, the experience of one who treats but a single fracture in a year is not considered nearly as conclusive as that of one whose cases are numbered by the hundreds, and yet much may be learned from a single fracture, especially if it is complicated and turns out badly.

"Failure to approximate the fragments means months of suffering to the patient, a prolonged convalescence, and perhaps permanent disability. Look at the tremendous task imposed upon nature when a fracture remains unreduced. The fibrinous material, instead of exuding between the fractured ends, as it would do were they in apposition and kept quiet, must bridge over the intervening space at a great disadvantage. The only wonder is that union takes place at all."

The next Sunday morning I read in the *New York Herald* of February 26, 1893, the following:

"Michael McMahon, thirty-two years old, a carriage-builder, of Fordham, went to the Manhattan Hospital five months ago, suffering from a broken jaw. Necrosis had set in, and the bone had to be scraped while the patient was under the influence of ether. McMahon was discharged, but returned to the hospital in December, still suffering from necrosis. His jaw refused to heal, and at his own request McMahon was again operated on last Wednesday. Visiting Surgeon William V. Wilkie performed the operation, assisted by several other physicians. He was examined, and the heart, kidneys, and liver were found in a perfectly healthy condition. Ether was administered at 4.30; the patient was back in bed, unconscious, at five o'clock, and despite all the efforts of the physicians, he died at a quarter past nine. One of the surgeons said last night, 'We did all we could for poor McMahon; why he died we do not know.' Deputy Coroner Weston declared that the man died of shock from ether."

This is a case that happens to a man once in a thousand times, and is always a surprise to the profession. Dr. Carl stated in his paper in September that he only wanted fifteen or twenty days to cure a patient. This patient was in the hospital for five months and was not cured.

DISCUSSION.

*Dr. Littig.*—In the case of those obturators, do you allow them to rest above the fissure or below?

*Dr. Bishop.*—Above. The case I gave you shows that the fissure is below. Talking brings these walls of the fissure up solid against the plate. The gentleman has worn it without any inconvenience for a long time. This can be inserted in three days. It took Dr. Stearns two days to make that little delicate spring that I showed you.

*Dr. Howe.*—I was rather surprised to hear Dr. Bishop speak so favorably of the operation of staphylorrhaphy, from the dentist's stand-point. Regarding as we do the question of the restoration of speech, I supposed that when clefts in the soft palate existed, and they were brought together by the operation of staphylorrhaphy, that there must necessarily be a permanent deficiency of tissue, and consequently imperfect speech. It is not a great many months since a patient was brought here who lately had a perforation of the soft palate, just at the posterior margin of the hard palate. The opening was said to have been no larger than would permit an ordinary-sized lead-pencil to pass through. It had healed spontaneously, and the gentleman was brought here to have some one tell why his speech had become imperfect since the lesion had healed. I suppose that is an illustration of what is likely to take place in every case where staphylorrhaphy is a surgical success. Will Dr. Bishop tell us what his ideas are on this point?

*Dr. Bishop.*—That is very true. Like all operations, the first few weeks or months, or possibly years, the cicatricial tissue is very rigid. The case I gave you in my paper last month, which was a case of rhinoplastic surgery, where the median line involved the right eye and half of the nose,—when that person was first operated on, the mouth was so rigid that he could hardly get food into it, but time softened it down. I cannot recall the surgeon who performed staphylorrhaphy, and was so disgusted that he afterwards took his knife and destroyed what he had done because he had not improved the voice; but if he had waited for a time I think there would have been a great improvement. This operation that I mentioned to-night was performed by Dr. David P. Smith, a professor in Yale College. He was surgeon on General Thomas's staff, and he performed it on a young man who was in Yale College. If we could only have a few months or a year for the patient to develop the parts united with the new tissue, I think we should get back the voice as well as though we had used an obturator, and would save the annoyance of an instrument. Of course, in a deformity of the mouth there is no instrument or operation that will make it absolutely perfect.

*Dr. Westlake.*—I appreciate the remarks of Dr. Howe in regard to the operation of staphylorrhaphy. I have heard similar reference made by some of our most eminent surgeons in New York, and they have all agreed that the dental profession has gained results when the operation of staphylorrhaphy has failed. With regard to the question of cicatricial tissue softening in time, I am pleased that such is the case. That muscles can be trained, I have no doubt.

In 1887 there was an operation—the excision of the inferior maxilla—reported in the *Dental Cosmos.* That operation was performed by Dr. McBurney at St. Luke's Hospital, where he was then surgeon. It was thought that by the application of a splint immediately after the operation the remaining jaw that was not injured could be kept in apposition, and it would be much easier to regulate the muscles on that side, and, in fact, all over the face. Dr. McBurney, through this case, has proven to other surgeons that it is necessary for the comfort of the patient and rapid healing, after an operation of that kind, to put a splint in immediately. That is where the dental profession has aided the surgical profession wonderfully. There has been no case ever reported where that difficulty has been overcome before. There is a cicatricial tissue where the excision was made at the angle of the ramus, which is very hard, and in nearly three years there has been no special softening of that tissue. The patient has had this apparatus (described in the *Dental Cosmos* in September, 1888) removed. The muscles were so trained that at the present time the patient has entirely overcome the retrograde movement. A lower denture is fitted in.

Dr. Bishop's valuable paper opened up new thoughts to me.

I question very much if the surgical profession will anticipate by the knife the benefit of well-adapted obturators. The operation of staphylorrhaphy, I believe, is obsolete, unless done very early in life.

*Dr. Ottolengui.*—The essayist, at the end of his paper, repeated the arguments of his predecessors, stating that we should not insert obturators in the mouths of children under fifteen years of age. I think, however, that I have seen the limit placed as low as twelve. I have come here to-night, attracted by the announced subject, to tell you what Dr. Kingsley has been doing of recent years for children between the ages of three and eight.

I pass around a model of a mouth of a child of six. Though so young, I will call her Miss S. When first seen she spoke so badly that only a little cousin, a playmate, could comprehend her meaning. She held no intelligible conversation with any one else, but communicated most of her wants by means of signs. That was eighteen months ago. Dr. Kingsley made an artificial soft palate, or velum, for her, and she was given a course of training by one who is a specialist in this sort of education. I saw the mother two months ago, and she informed me that whilst at Newport, last summer, little Miss S. was sent to school, and the teacher at that school called upon Mrs. S. to inquire what system of education had been given to this child which had enabled her to enunciate so well. There was no suspicion that there existed a deformity of any kind, but, on the contrary, it was remarked by this teacher, and by others, that the child's enunciation was uncommonly good.

In the next model you will observe that only the temporary teeth are present, little Miss C. being but five years of age. The temporary molar on one side was utilized after filling, but the fact that the molar upon the opposite side has been worn away to the gum line seemed to offer an insurmountable obstacle, as, of course, the plate which carries the velum must be attached to the teeth by clasps. Dr. Kingsley, however, suggested what I think has never been done before, and I crowned this temporary molar with gold,—no simple undertaking for a sensitive child of such tender years.

Dr. Kingsley then constructed his instrument, and about Christmas week of last year she was allowed to return to Boston. On January 1, or thereabout, the specialist before alluded to went on to Boston, and has been instructing the child daily ever since. In order that definite results might be proved, the child was made to talk into a phonograph, repeating a little child's poem which she had memorized, as she has not yet learned to read. When the phonograph repeats what was said at that time, one hears but a jumble of meaningless sounds. Her special training course will terminate on April 1, but already she has been made to repeat the same poem to the phonograph, and I am informed that the result now is quite intelligible, though of course, in time, and from a continuance of her daily exercises in enunciation, she will eventually speak more perfectly. This, however, shows how much may be gained in three months, when the patient is young, and before the formation of bad habits of abnormal speech. The people in her neighborhood are quite surprised to discover that the little girl has "overcome her stuttering," as they called it, and persons afflicted with stuttering have applied to the parents to know how they may be benefited.

A word on the subject of the soft palate, or velum. With a record like that attained in the above cases, we must admit that there is an inherent value to the velum not to be hastily overlooked. Many of the objections which have been urged are well founded. The soft rubber palate is often nasty, and frequently not durable; but I have observed that, like other things, it is nasty in the mouths of nasty people, whilst it may be quite clean in the mouths of the cleanly. The shortest duration for one I find to be six months; and as the cost is five dollars for each duplicate, which is about the charge for a pair of shoes which lasts no longer, it becomes a question whether it is not worth as much to talk well as to walk well. But I know one patient who has worn a palate continuously for ten years without needing a duplicate, whilst many last from two to five years.

After a person who is afflicted with *congenital* cleft palate has learned to speak with a velum, a hard rubber obturator may be substituted, and will serve all purposes. This, I may say parenthetically, explains why some dentists have given patients obturators in place of soft palates which they had previously worn, and then have unctuously reported that the patients "liked the obturators best." All the greater difficulties had been overcome by using the flexible instrument.

Looking into the fauces of these patients, the sides of the divided palate hang inactive. Let the patient attempt to swallow, and the sides approach each other. The velum allows this play and encourages it, so that in time a greater activity of the muscles may permit the sides to approach even closer together. An obturator simply plugs up the hole, and it is evident that the fissure would never lessen with such an instrument in place. Place an obturator in the mouth of a child, and in five years there would be a space all around the bulb, because the development of the child had naturally enlarged the cleft. But insert a soft palate, and the play permitted to the muscles will increase their mobility, so that the tendency towards enlargement, through development of the child, would be counterbalanced by the development of muscular activity, the cleft remaining as small as in the first instance. In proof of this assertion, I may state that a velum made for a child two years ago fits her to-day, and if any change is ever made, it will be to decrease the size of the flaps, to allow more play of the muscles.

As to staphylorrhaphy, the same rule holds. The earlier the operation is performed the better the result that may be hoped for.

There is a child who was brought to Dr. Kingsley at the age of three and a half years. The operation of staphylorrhaphy had been performed with exceptional success. Unfortunately, the anterior part reopened, but the posterior was so good that it seemed fair to give the child a chance to encourage the growth of the soft palate by inserting an obturator to close the opening in the hard. A little instrument was made for her,—a platinum clasp-plate carrying hard rubber,—and the usual course of instruction resulted in a marked improvement in speech. I hope that by this means the closed natural palate will acquire length and strength to perform its function.

*Dr. Littig.*—You mean that the instrument bridged the opening?

*Dr. Ottolengui.*—Yes, the hard rubber fitted over the opening and was held by the platinum plate. If the plastic operation had been a little more successful, and had closed the whole fissure, the child might have had a complete restoration, which would be due to the early operation.[1] It seems singular to sew up the harelip in

---

[1] Since making the above remarks, I have again seen the little patient for whom the obturator was made, and the present condition is interesting, as entirely refuting my hope in favor of staphylorrhaphy. When first seen, the posterior edge presented merely as a divided uvula, but with the development of the child during the three years which have elapsed, what was then seemingly a split uvula *is now a distinct cleft palate*, though of course smaller than it would have been had no operation been attempted. Nevertheless, it will necessitate the construction of a velum before perfect speech can be attained, so that the operation *which looked so much like a success three years ago must now be counted as a failure*, although there has been no tearing away of any of the cicatricial tissues. Another point of extreme interest is, that where the obturator was placed to cover the opening in the hard palate, the soft tissues along the edges of the fissure have grown so that they now nearly touch, and it is my belief that that opening could be easily closed by operation. This would be a benefit in simplifying the instrument needed and materially altering the tendency towards nasal resonance.—R. Ottolengui.

infancy and to leave the palate till the tenth year. If the surgeons must operate, let them do so early, and we may get some record which would be encouraging to staphylorrhaphy.

*Dr. Bogue.*—I would like to ask Dr. Ottolengui if I understood him right, that one of these models is from a case where the instrument was placed two years ago, has been worn since then, and that he has seen it since.

*Dr. Ottolengui.*—Yes.

*Dr. Bogue.*—Then you have seen that the opening continued to grow with the growth of the mouth?

*Dr. Ottolengui.*—Yes, I have observed that. But I said that where a soft palate was used this tendency was overcome, so that in remodelling the instrument the flaps will be made smaller, if anything, to allow sufficient play to the muscles.

*Dr. Littig.*—While staphylorrhaphy may be surgically a success, any attempt to bring the tissues together, according to my observation, has always been a failure, which I attribute to the secretions which are constantly forming in the nasal cavity and passing down, which prevents the healing of the parts at that point.

*Dr. Bogue.*—As time advances, and the individual continues to grow, the cleft would have naturally been larger. With the velum in place, and constantly worn, do you say that the cleft had actually diminished in size?

*Dr. Ottolengui.*—Not that it has diminished, but that it has not grown larger. Moreover, it is now able to close more closely, so that a smaller instrument will better serve.

*Dr. Bogue.*—That is the best speech on artificial vela that I have heard for the past ten years, and it seems to contain more of demonstrated truth. As you all know, I worked on that subject for eight or ten years. I certainly wish to express thanks to Dr. Ottolengui for the statements which he has made to-night and for his method of stating them. They must carry conviction to all who heard him and who have comprehended the principles that underlie operations for the relief of this defect.

I should like to say one thing further. I wanted to ask Dr. Bishop, How do you know? Upon what facts do you base an opinion? We fail to keep accurate records for years, and that is where the weight comes in these particular cases. Surgeons perform operations, and dental surgeons perform others, and after a few months, or perhaps even less than that, the patient disappears from their sight, and they and we never know the ultimate results that are reached. Years afterwards, those who come across cases of operations from staphylorrhaphy find no particular improvement. The principle advocated a few moments since, that the earlier those operations were made the more beneficial they are, I think, is true. I have seen one or two that seemed to be of some benefit, but I have never seen one patient operated on surgically that could speak perfectly.

A vote of thanks was offered to Dr. Westlake and Dr. Bishop for their interesting papers.

Adjournment.

John I. Hart, D.D.S.,
*Editor New York Odontological Society.*

